# Ischuria Renalis

**Published:** 1831-01-01

**Authors:** 


### XIII.

### REGIMENTAL HOSPITAL OF TANNAH.

[J. Bird, Esq. Reporter.]

### ISCHURIA RENALIS.

THIS is a very unfrequent, but a very dangerous disease. Many medical men, of great practice, have never once seen it—and when seen, it is not always that a post-mortem examination is obtained.

The patient, in the present instance, was an invalid of the garrison of Tannah, in the East Indies, aged 50, who was admitted into the Regimental Hospital, on the 18th October, 1818, complaining of paucity of urine—not more than a small tea-cupful in the 24 hours. He had nausea; but no fixed pain in any part. When first attacked, several hours before admission, he experienced some uneasiness in the lumbar region, attended with vertigo. He was ordered a dose of castor oil, and afterwards a draught with tinct. opii and nitrous ether. *Second day.* No alteration in the symptoms. The draught several times repeated. *Third day.* The sensorium appeared much affected, as marked by drowsiness, unsettled state of mind, and loss of memory. His eyes were suffused, yellow, and slightly injected—pulse slow and full—some convulsive motion in one of the arms. The catheter was introduced into the bladder, but no urine was found there. He was bled to twenty ounces—took four grains of calomel, with the same quantity of antimonial powder—and then had a blister to the lumbar region. The blood was cupped, and slightly buffed. *Fourth day.* Less affection of the sensorium—no increase of urine—a brisk purgative—blister to the nape of the neck. *Fifth day.* Sensorial disturbance increased—great drowsiness—tongue brown and hard—purgative medicines produced no effect. *Sixth day.* Coma continues to increase—bowels obstinately confined. Died on the seventh day.

*Dissection.* The stomach was much thickened, and its villous coat very vascular. The liver was enlarged—displaced the intestines, and had elongated the mesentery. The spleen was enlarged to five times its natural size, and was very firm. The colon was contracted throughout its whole extent. The kidneys were enlarged to double their usual size, and were much altered in structure, having the pelves unnaturally small, and the mamillary processes so changed as to be with difficulty recognized from the general mass of disease. The impression on Mr. Bird's mind was, that the urinary secretion must have been imperfect for a length of time. In the head, there was a general serous effusion throughout the substance of the brain, and into the lateral ventricles.*

The author is disposed to connect ischuria renalis with liver affection rather than organic disease of the kidneys. We are certainly inclined to attribute this dangerous malady more to a constitutional than to a local cause—more to a general disorder of function in various organs, than to an insulated organic disease, however extensive, of any one viscus. It is one of the various ways in which Nature breaks up the human fabric in despair, when her conservative powers are foiled!

---

### XIV.

### REGIMENTAL HOSPITAL, 13TH LIGHT INFANTRY.

### FATAL EPISTAXIS.

IN the fifth volume of the Calcutta Transactions, just received, we find the following report by Surgeon Seevewright.

*Case.* Corporal Ambler, aged 40, was admitted into hospital on the 28th September, 1826, in a state of profuse hæmorrhage from the nose, confined chiefly to the right nostril. It had commenced two hours previously, in his bar-

* Transactions of the Medical and Physical Society of Calcutta, Vol. V.

